# 3-(2-Hy­droxy­eth­yl)-1-(4-nitro­phen­yl)-1*H*-benzo[*d*]imidazol-3-ium bromide

**DOI:** 10.1107/S2414314624011684

**Published:** 2024-12-10

**Authors:** Halliru Ibrahim, Sizwe J. Zamisa, Muhammad D. Bala, Pinkie Ntola, Holger B. Friedrich

**Affiliations:** a Department of Chemistry, Durban University of Technology, PO Box 1334, Durban, 4000, South Africa; b School of Chemistry and Physics, University of KwaZulu-Natal, Private Bag X54001, Durban, 4000, South Africa; University of Aberdeen, United Kingdom

**Keywords:** crystal structure, benzoimidazolium salt, tetra­mer

## Abstract

The cation of the title salt, C_15_H_14_N_3_O_3_^+^·Br^−^, has a dihedral angle of 24.26 (6)° between its fused imidazole and 4-nitro­phenyl rings and the N—C—C—O torsion angle associated with the hy­droxy­ethyl substituent is 60.15 (17)°. In the crystal, the bromide ions act as double acceptors for hydrogen bonds from a hydroxyl group (O—H⋯Br) and a fused imidazolium moiety (C—H⋯Br). Additionally, C—H⋯O hydrogen bonds between the phenyl group and hydroxyl oxygen atom create a two-dimensional supra­molecular network extending diagonally in the crystallographic *bc* plane.

## Abstract 2

In the crystal of the title salt, the bromide ions act as double acceptors for hydrogen bonds from a hydroxyl group (O—H⋯Br) and a fused imidazolium moiety (C—H⋯Br). Additionally, C—H⋯O hydrogen bonds between the phenyl group and hydroxyl oxygen atom create a two-dimensional supra­molecular network extending diagonally in the crystallographic *bc* plane.

## Graphical abstract

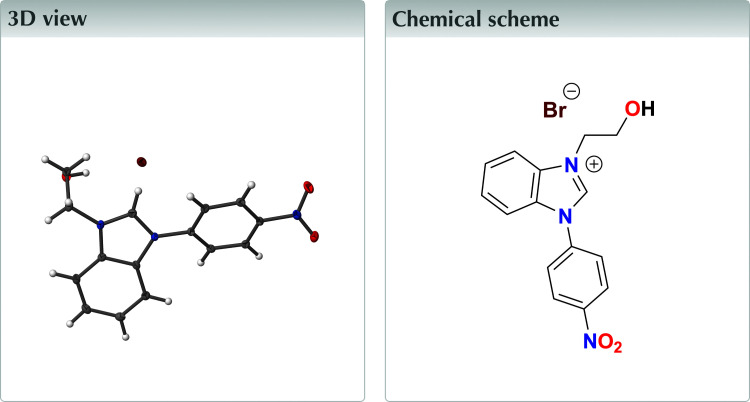

## Structure description

The title compound is a benzimidazolyl­idene precursor based on the 1-(4-nitro­phen­yl)benzimidazol-3-yl scaffold (Lee *et al.*, 2004 ▸; Ibrahim *et al.*, 2022 ▸) and quaternized to form a 2-hy­droxy­ethyl benzimidazolium bromide salt. Various works have reported the chemodosimetric potential of compounds with a fused 1*H*-benzo[*d*] backbone (Kumar *et al.*, 2013 ▸, 2015 ▸). The bulkiness of the backbone and the steric size of the ‘wingtip’ substituents influence the properties of such compounds in the absorption of nucleophiles such as cyanide ions. Their varied structures have led to investigations into their potential medicinal uses, thereby uncovering properties such as anti­microbial and anti­cancer activities (Kadafour *et al.*, 2022 ▸; Ott, 2017 ▸). Recently, we have focused on the development of imine-functionalized benzimidazolyl­idene compounds as potential ligands for earth-abundant metals that were utilized as homogeneous catalysts for the transfer hydrogenation of ketones (Abubakar & Bala, 2020 ▸; Kadafour & Bala, 2021 ▸). As part of our ongoing work aimed at developing new derivatives with enhanced catalytic properties, we synthesized the title compound, C_15_H_14_N_3_O_3_^+^ · Br^−^ (**I**), and determined its crystal structure.

**Figure 1:**
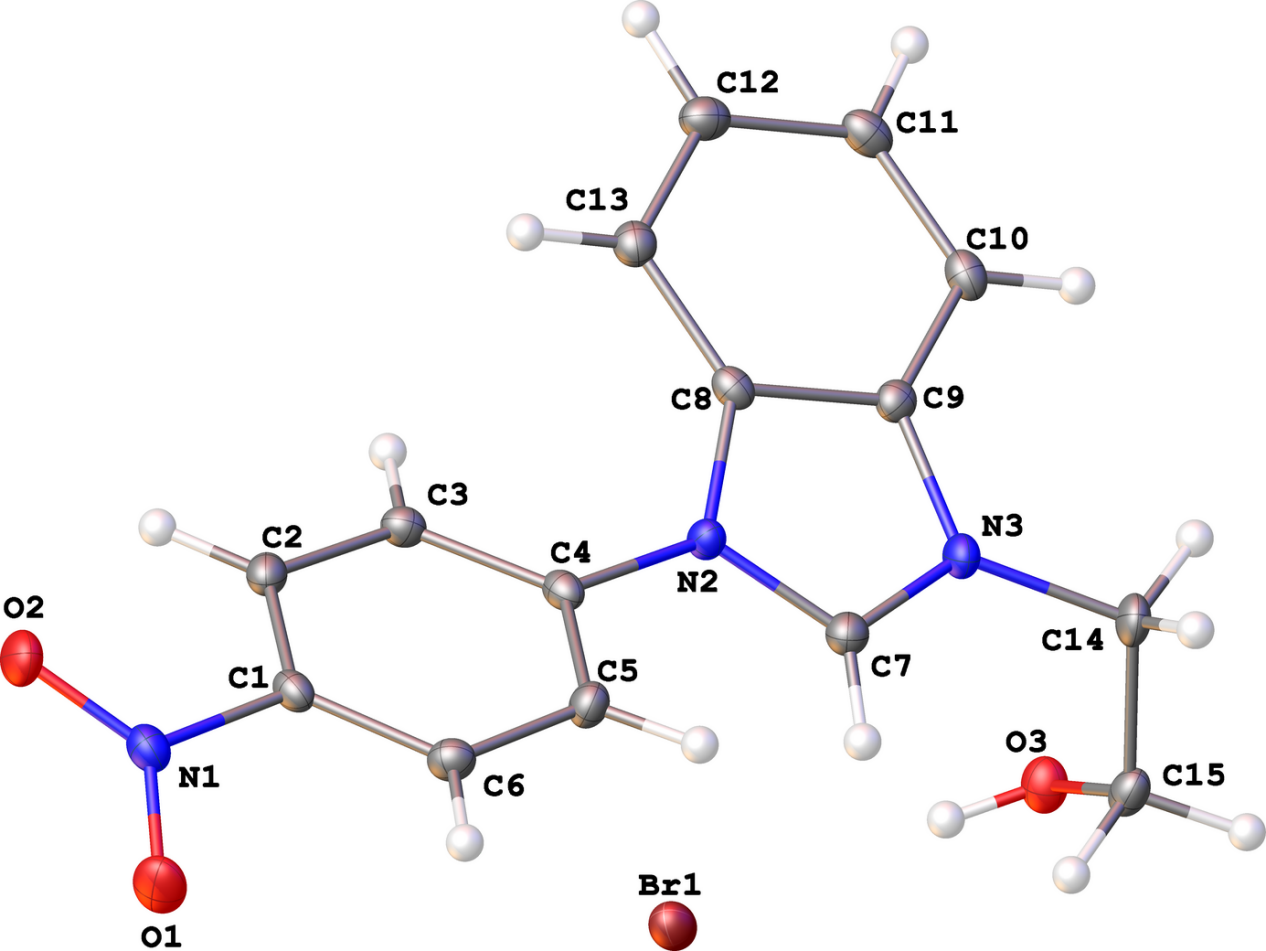
The mol­ecular structure of (**I**) showing displacement ellipsoids drawn at the 50% probability level.

**Figure 2:**
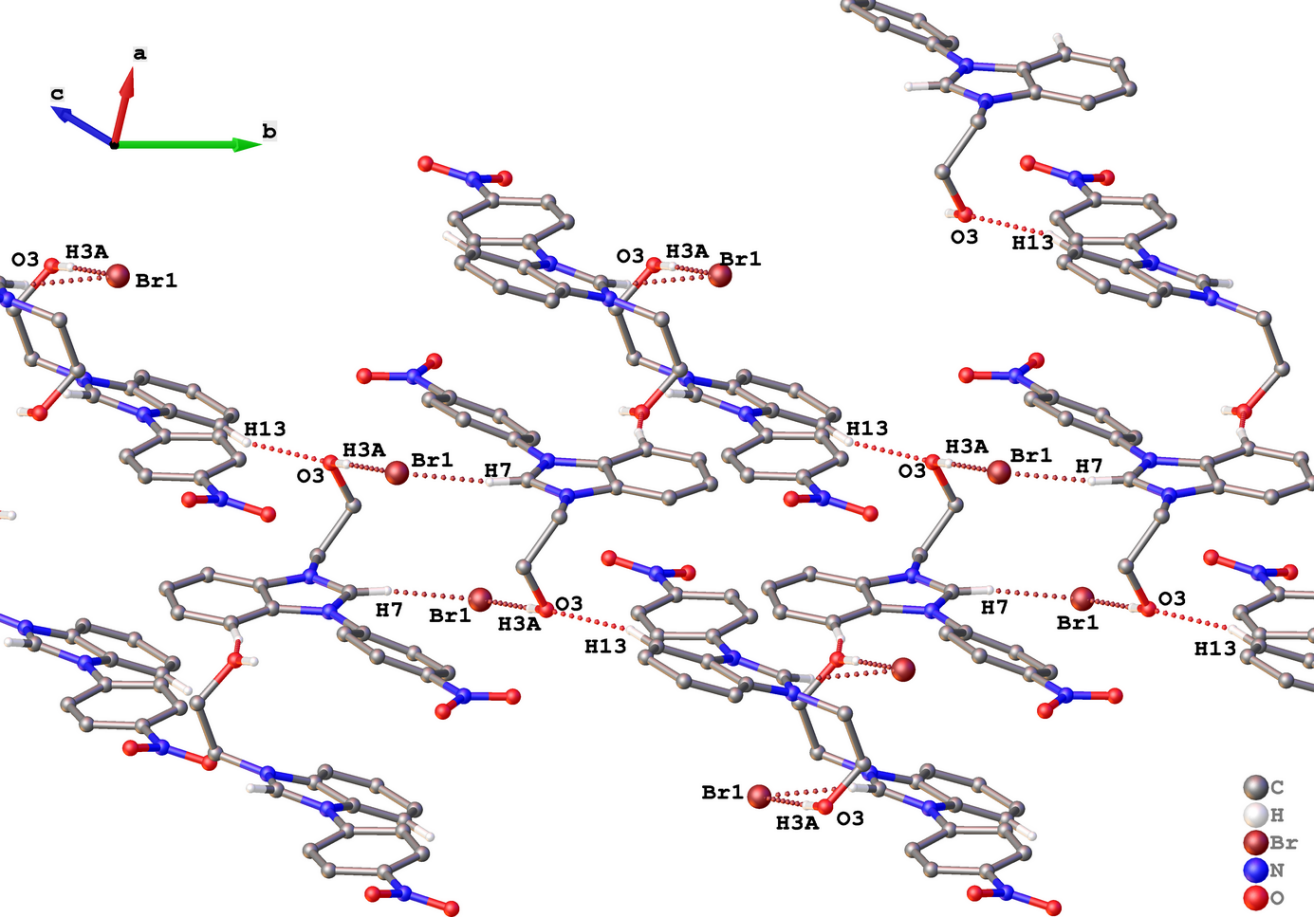
Representation of C7—H7⋯Br1, O3—H3*A*⋯Br1 and C13—H13⋯O3 hydrogen bonds (dotted bonds) in the packing of (**I**).

**Figure 3:**
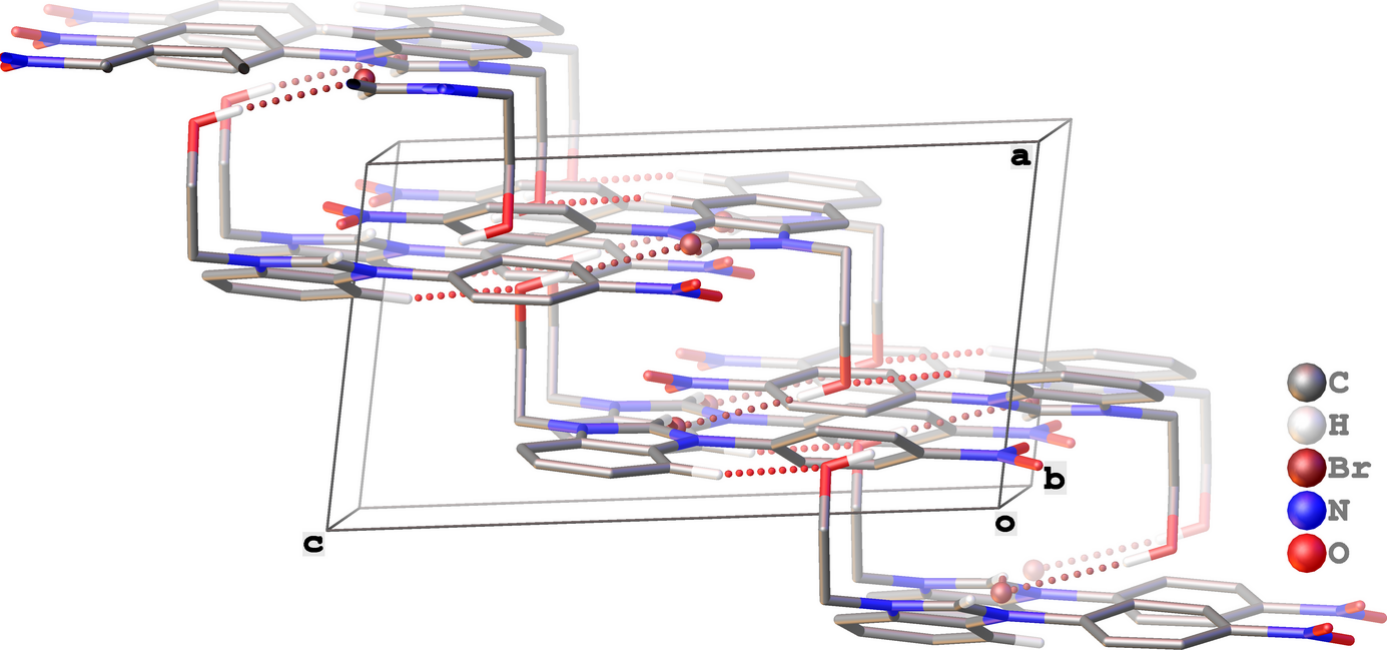
Representation of the propagation of the two-dimensional supra­molecular structure in (**I**).

The asymmetric unit of (**I**) consists of a cationic benzoimidazolium species and a bromide ion as depicted in Fig. 1 ▸. In comparison with the recently reported 3-(2-hy­droxy­eth­yl)-1-(4-nitro­phen­yl)-1*H*-imidazol-3-ium bromide (**II**) (Ibrahim *et al.*, 2024 ▸), the presence of the benzo­imidazole moiety in (**I**) seem to widen the dihedral angle between the imidazole and 4-nitro­phenyl rings from 8.99 (14)° in (**II**) to 24.26 (5)° in (**I**) while causing the ethanolyl side chain to adopt a synclinal conformation with respect to the fused imidazole ring [C7—N3—C14—C15 torsion angle = 59.7 (2)°]. In the extended structure of (**I**), the bromide ion acts as a double acceptor for O3—H3*A*⋯Br1 and C7—H7⋯Br1 links (Table 1 ▸) and inversion symmetry generates tetra­mers (two cations and two anions) with an 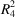
(16) graph-set descriptor, as shown in Fig. 2 ▸. Inter­molecular C—H⋯O hydrogen bonds exist between atom H13 of the phenyl moiety and O3 of the hy­droxy group (Fig. 2 ▸), which link the hydrogen-bonded 16-membered rings to form a two-dimensional supra­molecular structure that extends diagonally with respect to the crystallographic *bc* plane (Fig. 3 ▸).

## Synthesis and crystallization

The title compound was synthesized using a modified literature protocol (Ibrahim & Bala, 2016 ▸). To a Schlenk tube initially charged with *N-para* nitro­phenyl benzimidazole (0.50 g, 0.0021 mol) and an excess of 2-bromo­ethanol (0.78 g, 0.0063 mol) was added dry aceto­nitrile (20 ml). The mixture was stirred and refluxed under nitro­gen for 16 h. Removal of all volatiles from the greenish grey mixture and subsequent washing with batches of dry ethyl acetate (30 ml × 5) until the washing became colourless gave a grey solid, which was shown to be pure with TLC. The grey precipitate was then dried under vacuum to yield a greyish solid of the title compound. Colourless, block-shaped crystals of (**I**) suitable for crystal-structure determination were grown by the slow diffusion of diethyl ether into a methano­lic solution of the title compound. Yield: 0.42 g, 55.3%. m.p. 226–228°C. ^1^H NMR (400 MHz, DMSO-*d*_6_): δ_p.p.m._ 10.39 [*s*, 1H, NC(H)N], 8.67 (*d*, *J* = 8.9 Hz, 2 × 1H, CH_p_), 8.31 (*d*, *J* = 7.5 Hz, 1H, CH_b_), 8.21 (*d*, *J* = 8.9 Hz, 2 × 1H, CH_p_), 8.02 (*d*, *J* = 8.6 Hz, 1H, CH_b_), 7.87 (*m*, 2 × 1H, CH_b_), 5.29 (*s*, *b*, 1H, OH_e_), 4.74 (*t*, *J* = 9.8 Hz, 2H, CH_2 e_), 3.99 (*t*, *J* = 9.8 Hz, 2H, CH_2 e_): b = benzoyl, p = phenyl, e = ethanoyl. ^13^C NMR (100 MHz, DMSO-*d*_6_): δ_p.p.m._ 148.1 (NCN), 143.2, 138.1, 131.5, 130.7, 127.6, 127.1, 126.6, 114.5, 113.4, 58.6 (CH_2_), 50.1 (CH_2_). FTIR (cm^−1^): ν_O—H_ 3244; ν_aryl C—H_ 3081, ν_alkyl C—H_ 2997; ν_C=N_ 1566; ν_Nitro_ 1512, 1328; ν_C—O_ 1255;. LCMS (ESI^+^): *m*/*z* (%) 284.0635 (100) [(*M*—Br)]^+^.

## Refinement

Crystallographic data and structure refinement details are summarized in Table 2 ▸.

## Supplementary Material

Crystal structure: contains datablock(s) I. DOI: 10.1107/S2414314624011684/hb4498sup1.cif

Structure factors: contains datablock(s) I. DOI: 10.1107/S2414314624011684/hb4498Isup2.hkl

Supporting information file. DOI: 10.1107/S2414314624011684/hb4498Isup3.cml

CCDC reference: 2406833

Additional supporting information:  crystallographic information; 3D view; checkCIF report

## Figures and Tables

**Table 1:** Hydrogen-bond geometry (Å, °)

*D*—H⋯*A*	*D*—H	H⋯*A*	*D*⋯*A*	*D*—H⋯*A*
O3—H3*A*⋯Br1	0.84 (1)	2.39 (1)	3.2316 (11)	175
C7—H7⋯Br1^i^	0.95	2.68	3.5881 (16)	161
C13—H13⋯O3^ii^	0.95	2.39	3.3052 (19)	161

Symmetry codes: (i) 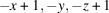; (ii) 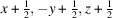.

**Table 2:** Experimental details

Crystal data	
Chemical formula	C_15_H_14_N_3_O_3_^+^·Br^−^
*M*_r_	364.20
Crystal system, space group	Monoclinic, *P*2_1_/*n*
Temperature (K)	100
*a*, *b*, *c* (Å)	6.7708 (1), 17.2107 (2), 12.3465 (2)
β (°)	98.184 (1)
*V* (Å^3^)	1424.09 (4)
*Z*	4
Radiation type	Mo *K*α
μ (mm^−1^)	2.90
Crystal size (mm)	0.32 × 0.19 × 0.13

Data collection	
Diffractometer	Bruker *SMARTAPEX2* CCD
Absorption correction	Multi-scan (*SADABS*; Krause et al., 2015 ▸)
*T*_min_, *T*_max_	0.628, 0.746
No. of measured, independent and observed [*I* > 2σ(*I*)] reflections	32857, 3555, 3006
*R*_int_	0.029
(sin θ/λ)_max_ (Å^−1^)	0.669

Refinement	
*R*[*F*^2^ > 2σ(*F*^2^)], *wR*(*F*^2^), *S*	0.022, 0.057, 1.04
No. of reflections	3555
No. of parameters	202
No. of restraints	1
H-atom treatment	H atoms treated by a mixture of independent and constrained refinement
Δρ_max_, Δρ_min_ (e Å^−3^)	0.38, −0.31

Computer programs: *APEX2* (Bruker, 2010 ▸), *SHELXT2013* (Sheldrick, 2015*a* ▸), *SHELXL2018/3* (Sheldrick, 2015*b* ▸) and *OLEX2* (Dolomanov *et al.*, 2009 ▸).

## full crystallographic data

### 3-(2-Hydroxyethyl)-1-(4-nitrophenyl)-1H-benzo[d]imidazol-3-ium bromide Crystal data

**Table:**

C_15_H_14_N_3_O_3_^+^·Br^−^	*F*(000) = 736
*M**_r_* = 364.20	*D*_x_ = 1.699 Mg m^−^^3^
Monoclinic, *P*2_1_/*n*	Mo *K*α radiation, λ = 0.71073 Å
*a* = 6.7708 (1) Å	Cell parameters from 9866 reflections
*b* = 17.2107 (2) Å	θ = 2.4–28.4°
*c* = 12.3465 (2) Å	µ = 2.90 mm^−^^1^
β = 98.184 (1)°	*T* = 100 K
*V* = 1424.09 (4) Å^3^	Rod, colourless
*Z* = 4	0.32 × 0.19 × 0.13 mm

### 3-(2-Hydroxyethyl)-1-(4-nitrophenyl)-1H-benzo[d]imidazol-3-ium bromide Data collection

**Table:**

Bruker SMART APEX2 CCD diffractometer	3006 reflections with *I* > 2σ(*I*)
Detector resolution: 7.9 pixels mm^-1^	*R*_int_ = 0.029
ω scans	θ_max_ = 28.4°, θ_min_ = 2.0°
Absorption correction: multi-scan (SADABS; Krause et al., 2015)	*h* = −9→9
*T*_min_ = 0.628, *T*_max_ = 0.746	*k* = −22→22
32857 measured reflections	*l* = −16→16
3555 independent reflections	

### 3-(2-Hydroxyethyl)-1-(4-nitrophenyl)-1H-benzo[d]imidazol-3-ium bromide Refinement

**Table:**

Refinement on *F*^2^	Primary atom site location: dual
Least-squares matrix: full	Hydrogen site location: mixed
*R*[*F*^2^ > 2σ(*F*^2^)] = 0.022	H atoms treated by a mixture of independent and constrained refinement
*wR*(*F*^2^) = 0.057	*w* = 1/[σ^2^(*F*_o_^2^) + (0.0263*P*)^2^ + 0.8714*P*] where *P* = (*F*_o_^2^ + 2*F*_c_^2^)/3
*S* = 1.04	(Δ/σ)_max_ = 0.001
3555 reflections	Δρ_max_ = 0.38 e Å^−^^3^
202 parameters	Δρ_min_ = −0.31 e Å^−^^3^
1 restraint	

### 3-(2-Hydroxyethyl)-1-(4-nitrophenyl)-1H-benzo[d]imidazol-3-ium bromide Special details

**Table:**

Geometry. All esds (except the esd in the dihedral angle between two l.s. planes) are estimated using the full covariance matrix. The cell esds are taken into account individually in the estimation of esds in distances, angles and torsion angles; correlations between esds in cell parameters are only used when they are defined by crystal symmetry. An approximate (isotropic) treatment of cell esds is used for estimating esds involving l.s. planes.

### 3-(2-Hydroxyethyl)-1-(4-nitrophenyl)-1H-benzo[d]imidazol-3-ium bromide Fractional atomic coordinates and isotropic or equivalent isotropic displacement parameters (Å^2^)

**Table:**

	*x*	*y*	*z*	*U*_iso_*/*U*_eq_	
Br1	0.24419 (2)	0.09433 (2)	0.50065 (2)	0.01679 (6)	
O1	0.83344 (19)	0.05748 (7)	1.03042 (9)	0.0227 (3)	
N1	0.84971 (18)	0.12439 (8)	0.99817 (10)	0.0149 (3)	
C1	0.8337 (2)	0.13769 (9)	0.88009 (12)	0.0126 (3)	
C2	0.7835 (2)	0.21094 (9)	0.84017 (12)	0.0134 (3)	
H2	0.766248	0.252648	0.888356	0.016*	
C3	0.7587 (2)	0.22218 (9)	0.72767 (12)	0.0129 (3)	
H3	0.716372	0.271150	0.697283	0.016*	
C4	0.7964 (2)	0.16110 (9)	0.66005 (12)	0.0120 (3)	
N2	0.77683 (18)	0.17216 (7)	0.54411 (10)	0.0114 (2)	
C7	0.7419 (2)	0.11328 (9)	0.47267 (13)	0.0138 (3)	
H7	0.723538	0.060653	0.492217	0.017*	
N3	0.73666 (18)	0.13869 (8)	0.37161 (10)	0.0131 (3)	
C14	0.7115 (2)	0.08907 (9)	0.27368 (13)	0.0165 (3)	
H14A	0.824064	0.051967	0.278145	0.020*	
H14B	0.714177	0.121769	0.207942	0.020*	
C15	0.5161 (2)	0.04420 (9)	0.26231 (12)	0.0152 (3)	
H15A	0.505858	0.010711	0.196555	0.018*	
H15B	0.515454	0.010147	0.326884	0.018*	
O3	0.34894 (17)	0.09455 (6)	0.25334 (9)	0.0177 (2)	
H3A	0.317 (3)	0.0968 (11)	0.3165 (6)	0.027*	
O2	0.87609 (17)	0.18096 (7)	1.05912 (9)	0.0203 (2)	
C8	0.7983 (2)	0.24063 (9)	0.48426 (12)	0.0117 (3)	
C13	0.8405 (2)	0.31742 (9)	0.51480 (12)	0.0139 (3)	
H13	0.863311	0.332851	0.589412	0.017*	
C12	0.8474 (2)	0.37011 (9)	0.43054 (13)	0.0159 (3)	
H12	0.874386	0.423144	0.448171	0.019*	
C11	0.8159 (2)	0.34784 (9)	0.31999 (13)	0.0155 (3)	
H11	0.820915	0.386053	0.264919	0.019*	
C10	0.7778 (2)	0.27144 (9)	0.28985 (12)	0.0140 (3)	
H10	0.757481	0.255874	0.215272	0.017*	
C9	0.7706 (2)	0.21846 (9)	0.37421 (12)	0.0119 (3)	
C5	0.8519 (2)	0.08802 (9)	0.70201 (12)	0.0139 (3)	
H5	0.880100	0.047268	0.654631	0.017*	
C6	0.8655 (2)	0.07536 (9)	0.81352 (12)	0.0141 (3)	
H6	0.895807	0.025258	0.843683	0.017*	

### 3-(2-Hydroxyethyl)-1-(4-nitrophenyl)-1H-benzo[d]imidazol-3-ium bromide Atomic displacement parameters (Å^2^)

**Table:**

	*U*^11^	*U*^22^	*U*^33^	*U*^12^	*U*^13^	*U*^23^
Br1	0.01940 (9)	0.01470 (9)	0.01707 (9)	0.00125 (6)	0.00532 (6)	0.00116 (6)
O1	0.0356 (7)	0.0170 (6)	0.0165 (6)	0.0021 (5)	0.0071 (5)	0.0052 (5)
N1	0.0148 (6)	0.0179 (7)	0.0125 (6)	−0.0002 (5)	0.0036 (5)	0.0007 (5)
C1	0.0119 (6)	0.0165 (8)	0.0097 (7)	−0.0021 (5)	0.0022 (5)	0.0008 (6)
C2	0.0131 (6)	0.0144 (7)	0.0130 (7)	−0.0016 (5)	0.0032 (5)	−0.0022 (6)
C3	0.0131 (6)	0.0121 (7)	0.0138 (7)	−0.0004 (5)	0.0025 (5)	0.0013 (6)
C4	0.0108 (6)	0.0155 (7)	0.0095 (7)	−0.0025 (5)	0.0010 (5)	0.0006 (6)
N2	0.0142 (6)	0.0107 (6)	0.0093 (6)	−0.0003 (5)	0.0015 (4)	0.0002 (5)
C7	0.0140 (7)	0.0140 (7)	0.0137 (7)	−0.0012 (5)	0.0029 (5)	−0.0011 (6)
N3	0.0150 (6)	0.0148 (6)	0.0095 (6)	−0.0013 (5)	0.0023 (5)	−0.0018 (5)
C14	0.0217 (8)	0.0179 (8)	0.0106 (7)	−0.0006 (6)	0.0041 (6)	−0.0044 (6)
C15	0.0215 (7)	0.0119 (7)	0.0119 (7)	0.0001 (6)	0.0015 (6)	−0.0017 (6)
O3	0.0214 (6)	0.0177 (6)	0.0139 (6)	0.0033 (4)	0.0021 (4)	0.0004 (5)
O2	0.0273 (6)	0.0216 (6)	0.0124 (5)	−0.0027 (5)	0.0044 (4)	−0.0037 (5)
C8	0.0098 (6)	0.0145 (7)	0.0109 (7)	0.0010 (5)	0.0018 (5)	0.0022 (6)
C13	0.0128 (7)	0.0156 (8)	0.0133 (7)	−0.0005 (6)	0.0019 (5)	−0.0009 (6)
C12	0.0151 (7)	0.0136 (8)	0.0194 (8)	−0.0006 (6)	0.0036 (6)	0.0009 (6)
C11	0.0144 (7)	0.0169 (8)	0.0159 (7)	0.0022 (6)	0.0042 (6)	0.0048 (6)
C10	0.0121 (6)	0.0192 (8)	0.0110 (7)	0.0016 (6)	0.0023 (5)	0.0007 (6)
C9	0.0104 (6)	0.0124 (7)	0.0130 (7)	0.0006 (5)	0.0021 (5)	−0.0010 (6)
C5	0.0162 (7)	0.0130 (7)	0.0125 (7)	−0.0001 (6)	0.0018 (5)	−0.0024 (6)
C6	0.0153 (7)	0.0125 (7)	0.0143 (7)	−0.0006 (5)	0.0013 (6)	0.0016 (6)

### 3-(2-Hydroxyethyl)-1-(4-nitrophenyl)-1H-benzo[d]imidazol-3-ium bromide Geometric parameters (Å, º)

**Table:**

O1—N1	1.2286 (18)	C14—C15	1.521 (2)
N1—C1	1.4644 (19)	C15—H15A	0.9900
N1—O2	1.2279 (17)	C15—H15B	0.9900
C1—C2	1.378 (2)	C15—O3	1.4172 (18)
C1—C6	1.387 (2)	O3—H3A	0.8399 (10)
C2—H2	0.9500	C8—C13	1.393 (2)
C2—C3	1.389 (2)	C8—C9	1.398 (2)
C3—H3	0.9500	C13—H13	0.9500
C3—C4	1.389 (2)	C13—C12	1.386 (2)
C4—N2	1.4314 (18)	C12—H12	0.9500
C4—C5	1.391 (2)	C12—C11	1.404 (2)
N2—C7	1.342 (2)	C11—H11	0.9500
N2—C8	1.4094 (19)	C11—C10	1.381 (2)
C7—H7	0.9500	C10—H10	0.9500
C7—N3	1.318 (2)	C10—C9	1.390 (2)
N3—C14	1.4703 (19)	C5—H5	0.9500
N3—C9	1.392 (2)	C5—C6	1.384 (2)
C14—H14A	0.9900	C6—H6	0.9500
C14—H14B	0.9900		
			
O1—N1—C1	118.16 (13)	C14—C15—H15B	109.3
O2—N1—O1	123.81 (13)	H15A—C15—H15B	107.9
O2—N1—C1	118.03 (13)	O3—C15—C14	111.79 (13)
C2—C1—N1	118.59 (13)	O3—C15—H15A	109.3
C2—C1—C6	123.15 (14)	O3—C15—H15B	109.3
C6—C1—N1	118.24 (13)	C15—O3—H3A	105.3 (14)
C1—C2—H2	120.9	C13—C8—N2	133.15 (13)
C1—C2—C3	118.26 (14)	C13—C8—C9	121.08 (14)
C3—C2—H2	120.9	C9—C8—N2	105.77 (13)
C2—C3—H3	120.3	C8—C13—H13	121.8
C4—C3—C2	119.31 (14)	C12—C13—C8	116.44 (14)
C4—C3—H3	120.3	C12—C13—H13	121.8
C3—C4—N2	120.11 (13)	C13—C12—H12	118.9
C3—C4—C5	121.57 (14)	C13—C12—C11	122.29 (15)
C5—C4—N2	118.31 (13)	C11—C12—H12	118.9
C7—N2—C4	122.63 (13)	C12—C11—H11	119.4
C7—N2—C8	107.97 (12)	C10—C11—C12	121.23 (14)
C8—N2—C4	129.33 (12)	C10—C11—H11	119.4
N2—C7—H7	124.8	C11—C10—H10	121.7
N3—C7—N2	110.49 (14)	C11—C10—C9	116.60 (14)
N3—C7—H7	124.8	C9—C10—H10	121.7
C7—N3—C14	124.76 (13)	N3—C9—C8	106.90 (13)
C7—N3—C9	108.87 (13)	C10—C9—N3	130.77 (14)
C9—N3—C14	126.26 (13)	C10—C9—C8	122.33 (14)
N3—C14—H14A	109.3	C4—C5—H5	120.4
N3—C14—H14B	109.3	C6—C5—C4	119.28 (14)
N3—C14—C15	111.62 (12)	C6—C5—H5	120.4
H14A—C14—H14B	108.0	C1—C6—H6	120.9
C15—C14—H14A	109.3	C5—C6—C1	118.27 (14)
C15—C14—H14B	109.3	C5—C6—H6	120.9
C14—C15—H15A	109.3		
			
O1—N1—C1—C2	158.13 (13)	C7—N2—C8—C9	1.00 (15)
O1—N1—C1—C6	−20.46 (19)	C7—N3—C14—C15	59.69 (19)
N1—C1—C2—C3	−176.82 (13)	C7—N3—C9—C8	0.33 (16)
N1—C1—C6—C5	−179.50 (13)	C7—N3—C9—C10	−179.36 (15)
C1—C2—C3—C4	−3.8 (2)	N3—C14—C15—O3	60.15 (17)
C2—C1—C6—C5	2.0 (2)	C14—N3—C9—C8	−176.11 (13)
C2—C3—C4—N2	−178.35 (12)	C14—N3—C9—C10	4.2 (2)
C2—C3—C4—C5	2.3 (2)	O2—N1—C1—C2	−20.94 (19)
C3—C4—N2—C7	−156.07 (14)	O2—N1—C1—C6	160.47 (13)
C3—C4—N2—C8	27.4 (2)	C8—N2—C7—N3	−0.83 (16)
C3—C4—C5—C6	1.5 (2)	C8—C13—C12—C11	−0.6 (2)
C4—N2—C7—N3	−178.02 (12)	C13—C8—C9—N3	178.45 (13)
C4—N2—C8—C13	−1.2 (3)	C13—C8—C9—C10	−1.8 (2)
C4—N2—C8—C9	177.94 (13)	C13—C12—C11—C10	−0.5 (2)
C4—C5—C6—C1	−3.5 (2)	C12—C11—C10—C9	0.6 (2)
N2—C4—C5—C6	−177.94 (13)	C11—C10—C9—N3	−179.76 (14)
N2—C7—N3—C14	176.82 (13)	C11—C10—C9—C8	0.6 (2)
N2—C7—N3—C9	0.32 (16)	C9—N3—C14—C15	−124.41 (15)
N2—C8—C13—C12	−179.21 (14)	C9—C8—C13—C12	1.8 (2)
N2—C8—C9—N3	−0.80 (15)	C5—C4—N2—C7	23.3 (2)
N2—C8—C9—C10	178.92 (13)	C5—C4—N2—C8	−153.20 (14)
C7—N2—C8—C13	−178.12 (16)	C6—C1—C2—C3	1.7 (2)

### 3-(2-Hydroxyethyl)-1-(4-nitrophenyl)-1H-benzo[d]imidazol-3-ium bromide Hydrogen-bond geometry (Å, º)

**Table:**

*D*—H···*A*	*D*—H	H···*A*	*D*···*A*	*D*—H···*A*
O3—H3*A*···Br1	0.84 (1)	2.39 (1)	3.2316 (11)	175
C7—H7···Br1^i^	0.95	2.68	3.5881 (16)	161
C13—H13···O3^ii^	0.95	2.39	3.3052 (19)	161

Symmetry codes: (i) −*x*+1, −*y*, −*z*+1; (ii) *x*+1/2, −*y*+1/2, *z*+1/2.
